# Nationwide handgrip strength values and factors associated with muscle weakness in older adults: findings from the Brazilian Longitudinal Study of Aging (ELSI-Brazil)

**DOI:** 10.1186/s12877-022-03721-0

**Published:** 2022-12-30

**Authors:** Bruno de Souza Moreira, Amanda Cristina de Souza Andrade, Juliana Lustosa Torres, Luciana de Souza Braga, Alessandra de Carvalho Bastone, Juliana Vaz de Melo Mambrini, Maria Fernanda Lima-Costa

**Affiliations:** 1 Núcleo de Estudos Em Saúde Pública E Envelhecimento, Universidade Federal de Minas Gerais E Fundação Oswaldo Cruz - Minas Gerais, Belo Horizonte, Minas Gerais, Brazil; 2 Programa de Pós-Graduação Em Saúde Coletiva, Instituto de Saúde Coletiva, Universidade Federal de Mato Grosso, Cuiabá, Mato Grosso Brazil; 3 Faculdade de Medicina, Programa de Pós-Graduação Em Saúde Pública, Universidade Federal de Minas Gerais, Belo Horizonte, Minas Gerais, Brazil; 4 Programa de Pós-Graduação Em Reabilitação E Desempenho Funcional, Universidade Federal Dos Vales Do Jequitinhonha E Mucuri, Diamantina, Minas Gerais Brazil; 5 Programa de Pós-Graduação Em Saúde Coletiva, Instituto René Rachou, Fundação Oswaldo Cruz, Belo Horizonte, Minas Gerais, Brazil

**Keywords:** Reference Values, Muscle Strength, Dynamometry, Weakness, Older Adults, Sarcopenia

## Abstract

**Background:**

Handgrip strength (HGS) is a simple, quick, inexpensive, and highly reliable method for the assessment of muscle strength in clinical practice and epidemiological studies. This study aimed at describing the HGS values by age group and sex in Brazilians aged 50 years and over, determining age group- and sex-specific cutoff points for muscle weakness, and investigating sociodemographic and anthropometric variables associated with muscle weakness for each sex.

**Methods:**

Data from the second wave of the Brazilian Longitudinal Study of Aging (ELSI-Brazil) were analyzed. HGS was measured in the dominant hand using a hydraulic hand dynamometer. Fractional polynomial regression models were fitted to estimate the percentiles (P5, P10, P20, P25, P50, P75, P90, and P95) of HGS by age group and sex. The P20 of the maximum HGS by age group and sex was used to define muscle weakness. Associations between sociodemographic (racial self-classification, place of residence, schooling, and monthly household income per capita in tertiles) and anthropometric variables (body mass index and waist circumference) and muscle weakness, by sex, were evaluated using logistic regression.

**Results:**

The analytical sample included 7905 participants (63.1 ± 9.1 years; 60% women). HGS reduced with increasing age in both sexes. Men presented higher HGS than women in all age groups. The cutoff points for muscle weakness ranged from 28 to 15 kg for men and from 17 to 9 kg for women. In the adjusted analyses, low schooling (0–4 years) was positively associated with muscle weakness in both sexes (in men, odds ratio (OR) 2.45, 95% confidence interval (CI) 1.46–4.12; in women, OR 1.90, 95%CI 1.18–3.06). Low and middle monthly household income per capita also had a positive association with muscle weakness among women (OR 1.78, 95%CI 1.37–2.32; OR 1.32, 95%CI 1.01–1.73, respectively). Overweight had a negative association with muscle weakness among men (OR 0.66, 95%CI 0.52–0.83), and obesity was inversely associated with muscle weakness in both sexes (in men, OR 0.49, 95%CI 0.31–0.78; in women, OR 0.69, 95%CI 0.52–0.92).

**Conclusions:**

This study provides HGS values and cutoff points for muscle weakness by age group and sex from a nationally representative sample of older Brazilian adults. The variables associated with muscle weakness slightly differed between men and women. HGS values and cutoff points generated can be used as benchmarks in clinical settings and foster future epidemiological research.

## Background

Muscle strength is an indispensable component for independence in daily living activities and healthy aging that presents a curvilinear relationship with age [1]. It means that muscle strength increases through childhood and adolescence up to reaching a peak in mid-adulthood (around the fourth decade of life) and thereafter declines remarkably in late life [1]. Individuals over 75 years of age exhibit approximately 60% less muscle strength compared to those aged 18–19 years [2].

Handgrip strength (HGS) is a simple, quick to administer, inexpensive, and highly reliable method [3] used to assess muscle strength in clinical settings and epidemiological studies. It has been recommended as a useful indicator for overall health, a vital sign, and a biomarker of health status [4]. Moreover, HGS is positively associated with the strength of other muscle groups, including lower limbs, which justifies its use as a proxy of overall muscle strength [5].

Prior work conducted in 125,462 healthy community-dwelling adults aged 35–70 years from 21 countries of all income strata found that HGS differs among the geographic regions and/or ethnic groups [6], which demonstrates the importance of country-specific reference values of HGS. Some studies using nationally representative samples have presented reference values of HGS for older adults in different countries, such as Colombia (≥ 60 years) [7], Portugal (≥ 65 years) [8], Ireland (≥ 50 years) [9], Singapore (≥ 60 years) [10], and United States (≥ 50 years) [11]. In Brazil, a multicenter study conducted in 16 Brazilian municipalities between 2009 and 2010, named Frailty in Brazilian Older People (in Portuguese, FIBRA) provided normative reference values of HGS for individuals aged 65–90 years [12]. However, this study was not nationally representative, the municipalities included were selected according to the convenience of the research coordinators, and only residents in urban areas were sampled. Additionally, previous research carried out with data from the Study of Chronic Diseases in Older People (in Portuguese, EDOC-I) conducted in the municipality of Rio Branco, northern Brazilian region, provided cutoff points for muscle weakness by sex and age group (60–69, 70–79, and ≥ 80 years) [13]. As far as we know, there is a lack of nationally representative Brazilian studies of older adults describing reference values of HGS and determining cutoff points for muscle weakness.

Establishing up-to-date reference values of HGS specific to the Brazilian population is essential to healthcare professionals, as these values can serve as benchmarks for comparing to the performance of their patients, which can lead to an improved physical-functional diagnosis and guide therapeutic approaches. In other words, these values can help in the identification of individuals at risk or with a deficit in muscle strength, who may benefit from preventive strategies or rehabilitation programs to promote the restoration of muscle strength to the expected values according to sex and age group. Furthermore, the cutoff points generated can foster future epidemiological research on muscle weakness and the odds of adverse events (e.g. disability, hospitalization, institutionalization, and mortality) in older adults.

To address gaps in existing knowledge, we analyzed HGS data from the Brazilian Longitudinal Study of Aging (ELSI-Brazil), a nationally representative survey of community-dwelling people aged 50 years and over. Thus, the goals of the present study were three-fold: (1) to describe HGS values for Brazilians aged 50 years and over stratified by age group and sex, (2) to determine age group- and sex-specific cutoff points for muscle weakness, and (3) to investigate sociodemographic and anthropometric variables associated with muscle weakness among men and women separately.

## Methods

### Study design

This cross-sectional study was conducted using data from the second wave of the ELSI-Brazil, the first wave of which was conducted in 2015–2016. To ensure that the sample represented the urban and rural areas of the small, medium, and large municipalities, the ELSI-Brazil adopted a multistage stratified cluster sampling design. The municipalities were allocated in four strata according to their population size. For the first three strata (municipalities up to 750,000 inhabitants), the sample was selected in three stages: municipality, census tract, and household. In the fourth stratum, which included the largest municipalities, the sample selection was performed in two stages: census tract and household. The drawing of households occurred in a systematic way, which consisted of a jump of four houses after an interview was carried out or after three unsuccessful contact attempts. The systematic jump was not performed in cases of refusal or ineligibility [(1) when there was no resident aged 50 years and over; (2) when the household was vacant; (3) when the household was collective (pension, asylum, republic, shelter, or hostel); (4) when the interviewee had some disability that prevented him/her from answering the questionnaire and there was no substitute informant (proxy)]. When the interviewer found any of these cases, he/she proceeded to the next household, following the right-hand rule. All residents aged 50 years and over in the selected households, including those with disabilities, bedridden, wheelchair users, were eligible for research. ELSI-Brazil wave 2 was conducted in 2019–2021, including participants from the previous wave plus replacement of the sample, to guarantee its national representativeness (n = 9949). Further details on the ELSI-Brazil’s sample and its national representativeness have been previously published [14, 15]. Other details can also be seen on the research homepage (http://elsi.cpqrr.fiocruz.br/en)**.** The Research Ethics Committee of the Oswaldo Cruz Foundation, Minas Gerais, approved the ELSI-Brazil protocol (CAAE: 34,649,814.3.0000.5091). Participants signed separate informed consent forms for the interviews and physical measurements.

### Data collection

#### Handgrip strength

HGS was measured by trained interviewers during the home visit using a hydraulic hand dynamometer with an adjustable handle (SAEHAN®, South Korea). The participants performed the test in a sitting position in an armless chair with the test arm held at the side of the body, elbow flexed at 90º, forearm in a neutral position (thumb up), and the wrist in a comfortable position. The mobile handle of the device was placed in the second position or adjusted, if necessary, according to the size of the participant's hand. The test was performed with the dominant hand and participants were instructed to squeeze the dynamometer handle as hard as possible for two seconds. Dominant hand was determined by asking participants if they were right- or left-handed. The examiner provided verbal encouragement during the test. After demonstrating the test to the respondent, three measurements were taken with a one-minute rest interval between each test. All readings were taken in kilograms (kg). The highest value among the three measurements was used in the current analyses. More details can be seen in the handbook on the survey homepage (http://elsi.cpqrr.fiocruz.br/en/questionnaires-and-interview-handbook/interview-and-physical-measurements-handbook/). In the current study, participants with HGS values lower than the 20^th^ percentile by age group and sex were considered as having muscle weakness [13].

#### Sociodemographic variables

Sociodemographic characteristics included racial self-classification (white or non-white e.g. black, brown, yellow, or indigenous), place of residence (urban or rural), schooling (0–4, 5–8, 9–11, or ≥ 12 years), and monthly household income per capita in tertiles (low, middle, or high). The urban–rural classification was based on the methods employed by the Brazilian Institute of Geography and Statistics (in Portuguese, IBGE) during the 2010 population census from administrative limits set out by local laws. Within each municipality’s boundaries, these local laws establish an imaginary line named “urban perimeter”. The census tracts located within the “urban perimeter” are termed urban, whereas the residual areas are designated rural. The other information was obtained through a face-to-face interview at the participant’s household.

#### Anthropometric variables

Height was measured in centimeters (cm) using a portable vertical stadiometer (NutriVida®, Brazil) with the participants barefoot with legs and feet parallel, weight distributed on both feet, arms relaxed at the sides, palms facing the body, and head in the Frankfurt horizontal plane. Weight was measured in kilograms (kg) using a portable digital scale (SECA®, Germany) with participants barefoot. Body mass index (BMI) was calculated as the ratio between weight in kilograms (kg) and height in square meters (m^2^). BMI cutoff points were based on the World Health Organization recommendation: underweight (< 18.5 kg/m^2^), eutrophic (18.5 to < 25.0 kg/m^2^), overweight (25.0 to < 30.0 kg/m^2^), and obese (≥ 30.0 kg/m^2^) [16]. Waist circumference was measured in centimeters (cm) using an inelastic measuring tape (SECA®, Germany). Participants were instructed to remain in a stand position, barefoot, with the blouse raised, feet apart, abdomen relaxed, and breathing normally. The measuring tape was positioned at the midpoint between the 10^th^ rib and the edge of the iliac crest. Measurements > 94 cm in men and > 80 cm in women were considered “increased” since they are associated with a higher risk of metabolic complications [17]. All anthropometric variables were measured twice during the home visit by trained interviewers and the mean of the measurements was used in the analyses. Further information can be seen in the handbook on the survey homepage (http://elsi.cpqrr.fiocruz.br/en/questionnaires-and-interview-handbook/interview-and-physical-measurements-handbook/).

### Statistical analysis

Firstly, descriptive characteristics of the study participants by sex were presented as percentages with their respective 95% confidence interval (CI). Additionally, we provided information on the most prevalent chronic diseases diagnosed by a physician in our sample. Secondly, fractional polynomial regression models were fitted to estimate the percentiles (P5, P10, P20, P25, P50, P75, P90, and P95) of HGS by age group and sex. This method provides smooth centile curves and explicit formulae for the percentile estimates. Diagnostic tests were used to evaluate the model fit (normal plot of the Z-scores, Shapiro–Wilk test, and residuals plot). The model was fitted by maximum likelihood, which accounts for non-normal skewness and/or kurtosis in the data [18]. Thirdly, HGS centile curves for men and women were plotted in charts. Finally, unadjusted and adjusted logistic regression models separate for men and women were created to investigate the sociodemographic and anthropometric variables associated with muscle weakness, being estimated the odds ratio (OR) with 95%CI. Variables with *p* < 0.20 in the unadjusted analyses were eligible for the adjusted analyses for each sex. The final model was composed of non-collinear sociodemographic and anthropometric variables associated with muscle weakness at *p* < 0.05. Due to possible collinearity between body mass index and waist circumference, we constructed adjusted models separately for these two variables. Then, the explanatory capacity of these models was compared using the Akaike Information Criterion (AIC), and the model with the best explanation was chosen. In addition, the adequacy of the adjusted models was evaluated using the F-adjusted mean residual goodness-of-fit test in which a non-significant result indicates a good adjustment. All analyses were carried out using the STATA software, version 16.0 (Stata Corporation, College Station, Texas, USA), accounting for the complex sample design and/or survey weights. The statistical significance level was set at 5%.

## Results

**Fig. 1:**
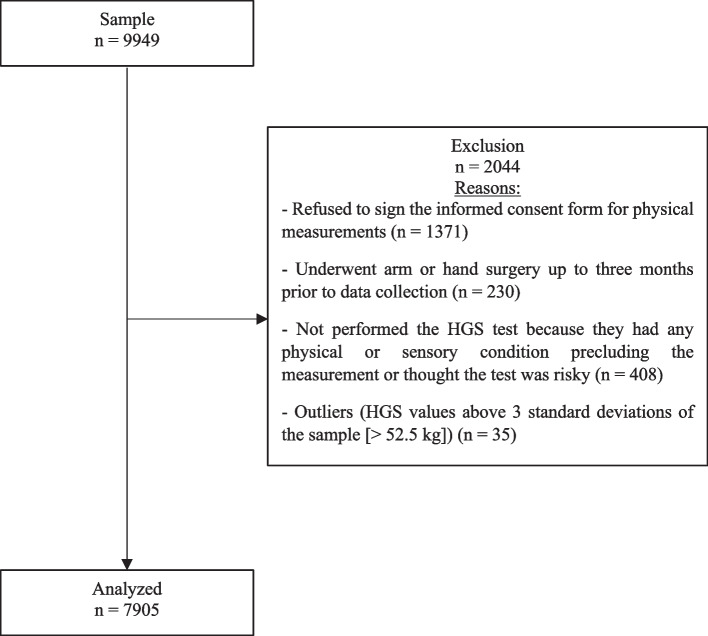
Study flow chart

Figure 1 describes the flow chart of the participants throughout the study. Our analytical sample was composed of 7905 participants aged 50–101 years (63.1 ± 9.1 years): 3162 men (63.2 ± 8.7 years) and 4743 women (63.1 ± 9.4 years). The excluding participants were similar to including participants regarding sex (*p* = 0.092), schooling (*p* = 0.813), and monthly household income per capita (*p* = 0.189) but tended to be older (≥ 80 years: 11.8% vs. 6.5%, respectively, *p* < 0.001).

The characteristics of the study participants by sex can be seen in Table 1. In both sexes, most participants were non-white, lived in the urban area, had 0–4 years of schooling, and presented increased waist circumference. Concerning BMI, overweight was the most frequent category for men (42.9%), while overweight and obesity had almost similar frequencies for women (36.9% and 35.4%, respectively). Regarding chronic diseases, 49.1% of participants reported having a medical diagnosis of arterial hypertension, 17.4% of diabetes mellitus, 11.5% of osteoporosis, 7.4% of heart diseases (angina, myocardial infarction, and heart failure), and 5.7% of respiratory diseases (asthma and chronic obstructive pulmonary disease).

**Table 1:** Characteristics of the study participants by sex. ELSI-Brazil, 2019–2021

Variables	Total	Men	Women
	**% (95% CI)**	**% (95% CI)**	**% (95% CI)**
**Sociodemographic**			
Racial self-classification^a^			
White	45.3 (39.1; 51.6)	45.1 (38.9; 51.5)	45.4 (38.8; 52.2)
Non-white	54.7 (48.4; 60.9)	54.9 (48.5; 61.1)	54.6 (47.8; 61.2)
Place of residence			
Urban	83.5 (77.1; 88.4)	83.9 (77.8; 88.6)	83.1 (76.3; 88.3)
Rural	16.5 (11.6; 22.9)	16.1 (11.4; 22.2)	16.9 (11.7; 23.8)
Schooling (years)^b^			
0–4	51.3 (47.4; 55.1)	50.8 (46.5; 55.1)	51.6 (47.4; 55.7)
5–8	20.7 (19.0; 22.6)	20.8 (18.8; 22.8)	20.7 (18.5; 23.2)
9–11	20.7 (18.4; 23.3)	22.0 (19.2; 25.0)	19.7 (17.2; 22.5)
≥ 12	7.3 (6.1; 8.7)	6.4 (5.2; 8.0)	8.0 (6.5; 9.8)
Monthly household income per capita in tertiles (R$)^c^			
Low	34.5 (29.7; 39.5)	33.0 (27.8; 38.6)	35.7 (30.8; 41.0)
Middle	33.2 (30.5; 36.0)	32.0 (29.0; 35.1)	34.2 (31.1; 37.4)
High	32.3 (28.2; 36.8)	35.0 (30.2; 40.3)	30.1 (26.2; 34.3)
**Anthropometric**			
Body mass index (kg/m^2^)^d^			
Underweight	2.0 (1.6; 2.5)	2.1 (1.6; 2.8)	1.9 (1.4; 2.5)
Eutrophic	28.0 (26.2; 29.9)	30.7 (28.3; 33.2)	25.8 (24.0; 27.8)
Overweight	39.6 (38.2; 41.0)	42.9 (40.9; 44.9)	36.9 (35.2; 38.6)
Obese	30.4 (28.9; 32.1)	24.3 (22.3; 26.5)	35.4 (33.4; 37.5)
Waist circumference (cm)^e^			
Adequate	25.4 (23.3; 27.7)	40.4 (37.3; 43.6)	13.2 (11.2; 15.6)
Increased	74.6 (72.4; 76.7)	59.6 (56.4; 62.7)	86.8 (84.4; 88.8)
**Number of participants (unweighted)**	7,905	3,162	4,743

*CI* confidence interval, *R$* reais

Missing data: ^a^21; ^b^62; ^c^266; ^d^133; ^e^443

Monthly household income per capita in tertiles: low (≤ R$ 750.00), middle (> R$ 750.00 to R$ 1250.00), and high (> R$ 1250.00 to R$ 40,000.00)

Body mass index: underweight (< 18.5 kg/m^2^), eutrophic (18.5 to < 25.0 kg/m^2^), overweight (25.0 to < 30.0 kg/m^2^), and obese (≥ 30.0 kg/m^2^)

Waist circumference: adequate (men ≤ 94 cm; women ≤ 80 cm) and increased (men > 94 cm; women > 80 cm)

All estimates considered the complex sample design and survey weights

**Fig. 2:**
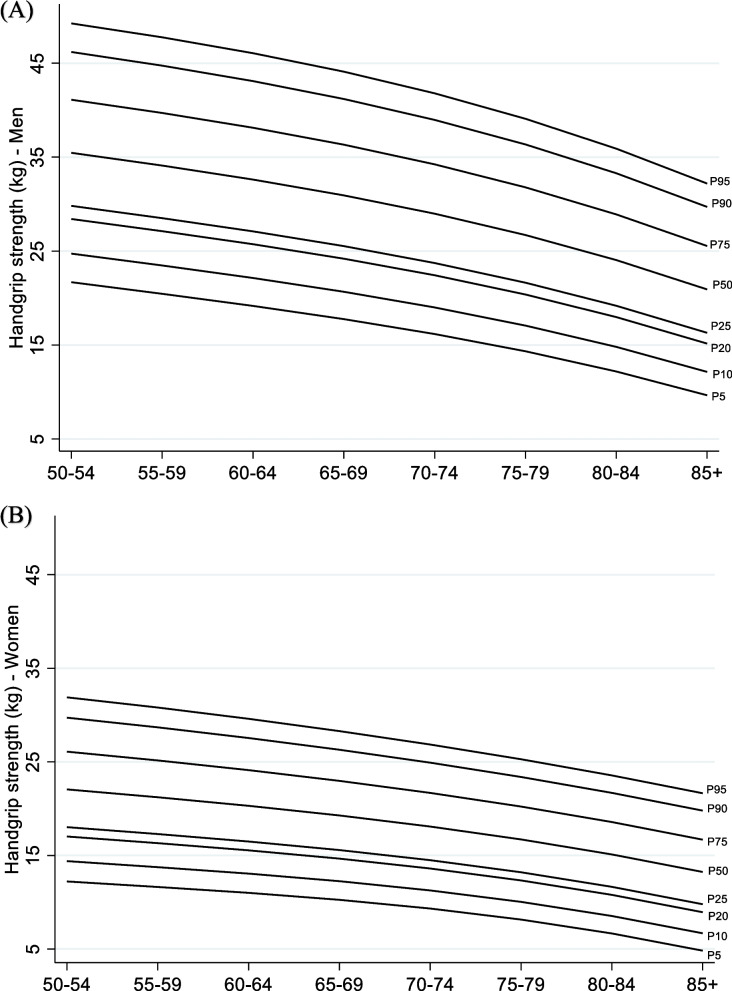
Handgrip strength centile curves for men (A) and women (B). ELSI-Brazil, 2019–2021

Table 2 presents the percentiles of HGS according to age group and stratified by sex and Fig. 2 illustrates the HGS centile curves for both sexes. There was a reduction in HGS values with increasing age in both sexes. The data also showed that men had higher HGS compared to women in all age groups. The 20^th^ percentile of HGS per age group in men ranged from 28 kg among participants aged 50–54 years to 15 kg among those aged 85 years and over. Regarding women, the 20^th^ percentile of HGS varied from 17 to 9 kg among those aged 50–54 and 85 years and over, respectively.

**Table 2:** Percentiles of handgrip strength (kg) according to age group and stratified by sex obtained through fractional polynomial regression. ELSI-Brazil, 2019–2021

Age in years by sex	*n*	P5	P10	P20	P25	P50 (M)	P75	P90	P95
**Men**									
50–54	312	22	25	28	30	35	41	46	49
55–59	657	20	23	27	29	34	40	45	48
60–64	611	19	22	26	27	33	38	43	46
65–69	535	18	21	24	26	31	36	41	44
70–74	401	16	19	22	24	29	34	39	42
75–79	317	14	17	20	22	27	32	36	39
80–84	188	12	15	18	19	24	29	33	36
≥ 85	141	10	12	15	16	21	26	30	32
Total	3162	16	19	23	25	31	37	42	45
**Women**									
50–54	525	12	14	17	18	22	26	30	32
55–59	919	12	14	16	17	21	25	29	31
60–64	847	11	13	16	16	20	24	28	30
65–69	825	10	12	15	16	19	23	26	28
70–74	639	9	11	14	14	18	22	25	27
75–79	499	8	10	12	13	17	20	23	25
80–84	319	7	9	11	12	15	19	22	24
≥ 85	170	5	7	9	10	13	17	20	22
Total	4743	9	11	14	15	19	23	27	29

*n* number of participants (unweighted), *P* percentile, *M* median

All estimates considered the survey weights

The unadjusted and adjusted associations of sociodemographic and anthropometric variables with muscle weakness for men and women are shown in Table 3. The adjusted models showed a positive association between low schooling (0–4 years) and muscle weakness in both sexes (in men, OR 2.45, 95%CI 1.46–4.12; in women, OR 1.90, 95%CI 1.18–3.06). The odds of muscle weakness were also significantly higher among women with low and middle monthly household income per capita (OR 1.78, 95%CI 1.37–2.32; OR 1.32, 95%CI 1.01–1.73, respectively). Overweight had a negative association with muscle weakness among men (OR 0.66, 95%CI 0.52–0.83), while obesity was inversely associated with muscle weakness in both sexes (in men, OR 0.49, 95%CI 0.31–0.78; in women, OR 0.69, 95%CI 0.52–0.92). Both models had a good adjustment as shown by the results of the F-adjusted mean residual goodness-of-fit test (model for men, *p* = 0.180; model for women, *p* = 0.946).

**Table 3:** Factors associated with muscle weakness^a^ for men (*n* = 3084) and women (*n* = 4551). ELSI-Brazil, 2019–2021

Variables	Men		Women	
	**Unadjusted**	**Adjusted**^**b**^	**Unadjusted**	**Adjusted**^**c**^
	**OR (95% CI)**	**OR (95% CI)**	**OR (95% CI)**	**OR (95% CI)**
**Sociodemographic**				
Racial self-classification (vs. white)				
Non-white	1.07 (0.82; 1.40)	-	1.15 (0.88; 1.49)	-
Place of residence (vs. urban)				
Rural	1.53 (1.02; 2.31)*	-	1.19 (0.76; 1.86)	-
Schooling (years) (vs. ≥ 12)				
0–4	2.70 (1.61; 4.51)**	2.45 (1.46; 4.12)**	2.52 (1.59; 4.01)**	1.90 (1.18; 3.06)*
5–8	1.72 (0.97; 3.05)	1.58 (0.88; 2.85)	1.86 (1.12; 3.09)*	1.46 (0.86; 2.47)
9–11	1.54 (0.83; 2.87)	1.53 (0.82; 2.87)	1.94 (1.19; 3.17)*	1.62 (0.98; 2.69)
Monthly household income per capita in tertiles (R$) (vs. high)				
Low	1.59 (1.07; 2.37)*	-	2.02 (1.55; 2.65)**	1.78 (1.37; 2.32)**
Middle	1.35 (0.92; 1.98)	-	1.48 (1.14; 1.94)*	1.32 (1.01; 1.73)*
**Anthropometric**				
Body mass index (kg/m^2^) (vs. eutrophic)				
Underweight	1.42 (0.77; 2.63)	1.42 (0.77; 2.62)	1.28 (0.67; 2.42)	1.08 (0.55; 2.14)
Overweight	0.63 (0.50; 0.79)**	0.66 (0.52; 0.83)**	0.92 (0.74; 1.14)	0.87 (0.71; 1.07)
Obese	0.46 (0.29; 0.71)**	0.49 (0.31; 0.78)*	0.70 (0.53; 0.93)*	0.69 (0.52; 0.92)*
Waist circumference (cm) (vs. adequate)				
Increased	0.58 (0.45; 0.75)**	-	1.04 (0.78; 1.38)	-

*OR* odds ratio, *CI* confidence interval, *R$* reais

^*^p < 0.05; **p < 0.001. F-adjusted mean residual goodness-of-fit test: ^b^*p* = 0.180; ^c^*p* = 0.946

^a^Muscle weakness was defined as values lower than the 20^th^ percentile specific for age group and sex (see values in Table 2)

Monthly household income per capita in tertiles: low (≤ R$ 750.00), middle (> R$ 750.00 to R$ 1250.00), and high (> R$ 1250.00 to R$ 40,000.00)

Body mass index: underweight (< 18.5 kg/m^2^), eutrophic (18.5 to < 25.0 kg/m^2^), overweight (25.0 to < 30.0 kg/m^2^), and obese (≥ 30.0 kg/m^2^)

Waist circumference: adequate (men ≤ 94 cm; women ≤ 80 cm) and increased (men > 94 cm; women > 80 cm)

All estimates considered the complex sample design and survey weights

^b^ Model adjusted for schooling and body mass index

^c^ Model adjusted for schooling, monthly household income per capita, and body mass index

## Discussion

The current study provides for the first time the age group- and sex-stratified values of HGS and cutoff points for muscle weakness based on a nationally representative sample of the community-dwelling Brazilian population aged 50 years and over. We found that HGS decreased with age in both sexes and men had higher HGS values than women in all age groups. Furthermore, the sociodemographic and anthropometric variables associated with muscle weakness slightly differed between the sexes. Among men, muscle weakness was positively associated with low schooling (0–4 years), and negatively associated with overweight and obesity. Among women, muscle weakness was positively associated with low schooling (0–4 years) and low and middle monthly household income per capita and negatively associated with obesity.

Considering the increase in life expectancy and the progressive growth of the older Brazilian population, it is expected that an expressive number of people will present a loss of muscle strength in the coming years. In this sense, our study contributes to the current body of literature providing up-to-date and reliable reference values of HGS. Furthermore, as muscle weakness defined by low HGS is used for the diagnosis of sarcopenia [19] and physical frailty [20], our results may be directly or indirectly useful for the research and management of these two geriatric syndromes among older adults in Brazil. Evidence shows that HGS mediates the relationship between muscle mass and frailty [21] and is a more useful single marker of frailty than chronological age alone [22].

As expected, our results showed that advancing age negatively affects HGS, which is in line with previous reports from different countries [7, 23–26]. Potential underlying mechanisms for this finding have been proposed in the literature. The aging process itself is associated with functional and structural modifications in multiple physiological systems, including musculoskeletal, nervous, vascular, and endocrine. These modifications lead to muscle strength decline due to reduction in muscle mass and impairment in muscle activation pattern (e.g. activation of agonist muscles decreases and coactivation of antagonist muscles increases) [27], as well as affect hand structures, such as joints, tendons, nerves, receptors, and blood supply [24, 26]. In addition, age-related chronic low-grade inflammation, characterized by increased levels of circulating cytokines (e.g. interleukin-6 and tumor necrosis factor-alpha), plays an important role in the loss of muscle strength that accompanies aging [28] by activating or blocking signaling pathways related to proteolysis and protein synthesis.

Accurate comparisons of our results with data from other five studies with nationally representative samples are hampered by methodological heterogeneity between studies [7–11]. In general, these differences refer to the number of measurements, hand examined (right and/or left, dominant and/or non-dominant), and use of the highest or mean value of the measurements. In addition, two studies considered height in addition to the age group in determining the HGS reference values for men and women [8, 9].

In the Brazilian scenario, five studies provided reference values of HGS for older adults [12, 13, 29–31]. No study used a nationally representative sample and four of these studies were carried out with individuals from only one municipality [13, 29–31]. Again, great difficulty was observed in comparing our results with those described in these studies because of methodological differences. Some authors presented the HGS values for each sex stratifying the age group in decades [13, 29] and other authors in 5-year intervals different from the present study [31]. There were also different categorizations of the oldest age group: ≥ 70 [29]; ≥ 76 [31]; ≥ 80 years [13]. It is also worth noting that all Brazilian studies differed in terms of how to obtain the HGS variable considering factors such as the number of measurements, hand examined, and use of the highest or mean value of the measurements. These methodological differences reinforce the urgent need for a consensus for standardization on how to measure HGS in order to improve data comparison in the future.

In recent years, a growing number of studies have determined cutoff points for muscle weakness (i.e. low HGS) for older populations [7, 13, 32–34]. For instance, Lera et al. established cutoff points for muscle weakness in a sample of 5250 Chileans aged 60 years and over based on the 25^th^ percentile (≤ 27 kg for men and ≤ 15 kg for women) [32]. In a study conducted in 1317 participants aged 60 years and over from China, Dong et al. determined cutoff points for muscle weakness associated with mobility limitation (walking speed < 0.8 m/s), with HGS values < 32.45 kg and < 18.20 kg for men and women, respectively [33]. Another different method for determining HGS cutoff values is calculating the mean minus 2.5 standard deviations of young healthy population [34]. Similarly to our study, Ramírez-Vélez et al., using nationally representative data from the Survey on Health, Well-Being and Aging in Latin America and the Caribbean (SABE), defined cutoff points for muscle weakness specific for age group and sex for Colombian older adults aged 60 years and over [7]. All cutoff points in the present study were higher when compared to those described for the older Colombian population. Potential reasons for this divergence between studies include heterogeneous methods for calculating the cutoff points and differences in participants’ genetic and clinical characteristics.

Our study demonstrated that low schooling was significantly associated with increased odds of muscle weakness in both sexes. Such association was also observed in previous national and international studies [35–37]. It is well known that individuals with higher educational attainment have more access to health services and health knowledge, adopt healthier lifestyle habits in terms of nutrition and physical activity, experience lower rates of unemployment, and earn a higher income, all of which might affect the overall health and also muscle strength [35]. In this sense, it is recommended that education and adequate information on healthy eating and physical exercise should be offered to older adults with a low educational level in order to prevent, postpone, or reverse muscle weakness.

This study also identified monthly household income per capita as a determinant of muscle weakness among older women in Brazil. Women with a low and middle income had 1.78 and 1.32-fold increased odds of muscle weakness, respectively, when compared to women with high income. A possible explanation for the observed association is that income affects individuals’ health outcomes in several ways, including by means of the availability of material resources and health services [36]. For example, limited financial resources might reduce access to adequate diet and nutrients (e.g. protein intake) and rehabilitation services, especially among the poorest individuals. By contrast, a previous large-population survey conducted in Korean participants aged 65 years and over found no significant association between income and muscle weakness in both sexes [35]. The authors argued that the income may not fully represent resources available at old age, mainly after retirement, and suggested that wealth could be a better measure of financial resources [35]. Similarly, research conducted in 27,351 participants aged 50 years and over from the Survey of Health, Ageing and Retirement in Europe (SHARE), involving 11 countries, showed that wealth has a greater impact on HGS than income [38]. Thus, further studies to examine the relationship between income or wealth and muscle weakness, particularly in developing countries, are warranted to draw conclusions that are more robust on this issue.

Another remarkable result of the present study is related to BMI. Overweight and obesity had a protective effect for muscle weakness when compared to BMI’s normal range (eutrophy). This observation is in agreement with a recent cross-sectional study conducted in 3342 participants aged 60 years and over from the Irish Longitudinal Study on Ageing (TILDA) that found an inverse relationship of probable sarcopenia (defined as HGS < 27 kg for men and HGS < 16 kg for women) with both overweight and obesity [39]. Recently, researchers also observed that when obesity accompanies probable sarcopenia, it might be less detrimental in terms of frailty and physical performance, compared to probable sarcopenia without increased adiposity [40]. In addition, the literature reports a U-shaped relationship between BMI and all-cause mortality in older adults, with the BMI ranges representing overweight and class I obesity being advantageous for survival [41]. According to Turusheva et al. [42], obese individuals have greater muscle mass and more type IIb muscle fibers (fast-twitch, glycolytic), which can lead to higher HGS. On the other hand, a Finnish longitudinal study demonstrated that being overweight or obese at baseline predicted greater HGS decline over a 22-year follow-up [43]. The researchers concluded that a lengthened duration of obesity can lead to muscle strength decline by means of inflammation and insulin resistance, which have catabolic effects on muscles [43]. Other studies found no significant association between HGS and BMI in older men and women in the multivariate analyses [24, 25]. Therefore, the relationship between these variables calls for further examination.

Unlike several previous studies conducted with healthy cohorts, we chose to keep individuals with chronic diseases in our analyses. It is noteworthy that identifying a healthy cohort (i.e. separating pathological from physiological age-related changes) is a hard task [12] and removing all participants with chronic diseases could generate a small and highly atypical sample [9]. In addition, the exclusion of participants with chronic diseases could reduce statistical power and affect the representativeness of our sample, resulting in a significant bias in the results.

The main strength of this work includes data analysis of a large sample derived from a nationwide-based study with rigorous sampling plans, data-collection procedures, and quality-control practices, which enhances the generalizability of our findings and strengthens the statistical reliability of the results. Another advantage refers to the use of a dynamometer, which made it possible to objectively and reliably evaluate muscle strength. It is also worth mentioning that, in the present study, we chose to consider the age group in determining the cutoff points for muscle weakness for both sexes, due to the widely known impact of age on HGS. The adoption of a single cutoff point for each sex, regardless of age, could lead to misleading findings, as a small number of participants in the younger age groups would be classified as having muscle weakness and a high proportion of participants in the oldest age groups would be classified as weak [13].

On the other hand, our findings should be interpreted in light of some limitations. First, our HGS data are cross-sectional and are likely to underestimate individual decline. Thus, curves and percentiles generated should not be used to monitor the trajectory of an individual’s muscle strength over time. There is also a possibility of our findings being influenced by differences in the birth cohort. Second, the exclusion of a higher proportion of older participants tends to underestimate the force of associations. Third, the sample of participants aged 85 and over was small, which prevented stratification and assessment of HGS among nonagenarians. Fourth, the associations of muscle weakness with important anthropometric variables, such as upper arm circumference, arm length, calf circumference, and hand size were not tested, because these variables were not collected in the ELSI-Brazil study. Finally, the method used to determine the cutoff points for muscle weakness was based on the 20th percentile of HGS according to sex and age group. The optimum methods to define cutoff points for muscle weakness include anchoring these cutoff points to any meaningful outcome (e.g., mobility limitation or disability) or determining mean minus 2.5 standard deviations of young and healthy individuals of a given population. Thus, additional research should be carried out to determine these cutoff points for the older Brazilian population using the aforementioned methodological approaches.

## Conclusions

This study extends the existing knowledge by providing reference values of HGS for Brazilians aged 50 years and over living in the community. Our results demonstrated that HGS varies according to age group and sex, which reinforces the importance of considering different values for these groups in clinical and research settings. The cutoff points generated should be used by healthcare professionals to recognize weak or at-risk people who would benefit from early interventions or rehabilitation programs to preserve or restore muscle strength to avoid functional limitations.

## Data Availability

The data that support the findings of this study are available in the ELSI-Brazil’s repository [http://elsi.cpqrr.fiocruz.br/en/register/] but restrictions apply to the availability of these data, which were used under license for the current study, and so are not publicly available. Data are however available from the authors upon reasonable request and with permission of the ELSI-Brazil’s coordination.

## Abbreviations

AIC: Akaike Information Criterion
BMI: Body mass index
CI: Confidence interval
EDOC-I: Study of Chronic Diseases in Older People
ELSI-Brazil: Brazilian Longitudinal Study of Aging
FIBRA: Frailty in Brazilian Older People
HGS: Handgrip strength
IBGE: Brazilian Institute of Geography and Statistics
OR: Odds ratio
SABE: Survey on Health, Well-Being and Aging in Latin America and the Caribbean
SHARE: Survey of Health, Ageing and Retirement in Europe
TILDA: Irish Longitudinal Study on Ageing
